# Inertial Focusing and Separation of Particles in Similar Curved Channels

**DOI:** 10.1038/s41598-019-52983-z

**Published:** 2019-11-12

**Authors:** Yue Ying, Ying Lin

**Affiliations:** School of Mechanical Engineering, Shanghai Institute of Technology, Shanghai, 201418 China

**Keywords:** Mechanical engineering, Fluid dynamics

## Abstract

Inertial particle focusing in curved channels has enormous potential for lab-on-a-chip applications. This paper compares a zigzag channel, which has not been used previously for inertial focusing studies, with a serpentine channel and a square wave channel to explore their differences in terms of focusing performance and separation possibilities. The particle trajectories and fluid fields in the curved channels are studied by a numerical simulation. The effects of different conditions (structure, Reynolds number, and particle size) on the competition between forces and the focusing performance are studied. The results indicate that the zigzag channel has the best focusing effect at a high Reynolds number and that the serpentine channel is second in terms of performance. Regarding the particle separation potential, the zigzag channel has a good performance in separating 5 μm and 10 μm particles at *Re*_*C*_ = 62.5. In addition, the pressure drop of the channel is also considered to evaluate the channel performance, which has not been taken into account in the literature on inertial microfluidics. This result is expected to be instructive for the selection and optimization of inertial microchannel structures.

## Introduction

The development of inertial microfluidics^1^ has enabled particle manipulation with improved performance over sheath flow control^2^ and external force generators^3^ and has made it possible to miniaturize the devices and simplify the operation^4^. Segre and Silberberg^5^ reported the inertial migration of particles in a circular tube in the 1960s, which first brought inertial microfluidics to the attention of researchers. Due to its many advantages, inertial microfluidics has been widely employed for the focusing^6^, separation^7^, and fractionation of bioparticles and has been used in other fields. Steerable objects have become more diverse to accommodate particles of different biophysical characteristics (e.g., size^8^, density, shape^9^, and deformability) and to precisely control these particles. In addition to straight channels, there are three basic types of microchannel structures: expansion-shrink array channels, spiral channels and serpentine channels. These structures were created to introduce extra inertial effects, such as Dean flow, to achieve more precise manipulation of particles. Among these channel structures, the serpentine channel is arousing the interest of an increasing number researchers because of its small footprint and simple parallelization and the fact that the processing efficiency can be multiplied. The flow pattern in a serpentine channel is more complicated than that in a straight or spiral channel due to the alternating curve geometry. This phenomenon causes the particles to swing along the channel centerline, which is expected to provide an effective means of achieving precise passive particle manipulation.

As early as 2007, Di Carlo^4^ adopted symmetric and asymmetric serpentine channels to achieve particle focusing in a pioneering work of inertial microfluidic control. Gossett *et al*.^10^ found that the channel curvature can result in reduced fluid resistance for microfluidic designs and can be used for low-flow inertial focusing or separation. Toner *et al*.^11^ employed serpentine channel particle focusing as a pretreatment step for magnetic separation technology, greatly improving the magnetic separation efficiency. Wang *et al*.^12^ achieved focusing and separation of submicrometer particles in a serpentine channel. However, the results of particle-size-dependent separation in asymmetric serpentine channels are not ideal^13^. Instead, many researchers have realized the separation of particles of different sizes in a symmetric serpentine channel. Zhang *et al*.^14^ reported a comprehensive study of the channel structure parameters and flow conditions for inertial particle focusing in symmetrical sinusoidal/serpentine channels. Obzey *et al*.^15^ studied a serpentine channel with a curvature angle of 280° to elucidate the basic physical properties of the particle dynamics. In addition to the conventional serpentine channel, researchers also conducted a series of fundamental and applied studies on a square wave channel, which was also named as a serpentine channel in their works. In terms of fundamental mechanisms, Zhang *et al*.^16^ provided insights into the role of the centrifugal force in inertial focusing and demonstrated for the first time that a single focusing streak can be achieved in a symmetric serpentine channel. Then, the authors achieved the separation of particles of different sizes based on the difference in the particle lateral focusing positions^17^. In biomedical applications, Zhang’s group^18^ successfully separated blood cells from plasma with a purity of 99.95% and developed a continuous high-throughput microfluidic WBC separation platform. The purity of the WBCs could be increased to 48%^19^. In addition, the interaction of the inertial lift force and dielectrophoretic force in a square wave channel was studied^20^, and a tunable separation method was proposed by an innovative hybrid DEP-inertial microfluidic platform^21^. All of these studies investigated the application of curved channels in inertial particle focusing, but it should be noted that the above experimental studies could not adequately study the interaction between particles and flows by showing the focusing behavior of the particles. This problem can be solved very well via a numerical simulation. Rasooli *et al*.^22^ developed a Lagrangian model using COMSOL Multiphysics to solve the continuous phase and simulate particle trajectories in a spiral microchannel. Jiang *et al*.^23^ explored the particle focusing mechanisms of a symmetric serpentine microchannel based on a lattice Boltzmann method. Although the study of inertial focusing in serpentine channels has been successful, the difference in focusing effects in similar curved channels is still unknown.

This paper aims to study the difference in particle focusing effects and separation possibilities in similar curved channel geometries. A zigzag channel, which has not been used previously for inertial focusing studies, is introduced with the serpentine channel and square wave channel to conduct research by numerical simulations. First, a simulation model is verified based on previous experimental results. Then, the particle trajectories and fluid fields are obtained to analyze the particle migration behaviors by considering the competition between the inertial lift forces and Dean drag forces on the particles. Next, the particle focusing effects are investigated by varying the particle size and Reynolds number to determine the desirable focusing conditions. Finally, the separation of particles of two sizes is attempted to explore the functionalities of curved channels.

## Working Principle

Since the first application of inertial effects in microfluidics in 2007, many studies have explained the physics of inertial effects in the literature. Particles in a microchannel are subjected to an external force called an inertial lift force (*F*_*L*_), which consists of a shear gradient lift force (*F*_*S*_) and a wall-induced lift force (*F*_*W*_). In Poiseuille flow, due to the curvature of the fluid velocity profile and its interaction with particles, the shear gradient lift force directs particles away from the channel center. As the particles move towards the channel walls, due to the flow field interaction between the particles and the walls, the wall-induced lift force directs particles away from the walls. These opposite lift forces compete with each other over the channel cross-section. When the lift forces in the opposite directions are equal, particles tend to occupy equilibrium positions and form narrow bands. Previous studies have reported critical conditions for achieving inertial focusing: $\lambda ={{a}}_{{P}}/{{D}}_{{h}} > 0.07$ and ${R}{{e}}_{{P}}={R}{{e}}_{{C}}\times {({{a}}_{{p}}/{{D}}_{{h}})}^{2} > 1$^4^, where *a*_*p*_ is the diameter of the particle, *D*_*h*_ is the hydraulic diameter of the channel, and *Re*_*P*_ and *Re*_*C*_ are the Reynolds numbers of the particle and channel, respectively.

The net inertial lift force was derived by Asmolov^24^ and then simplified as follows^25^

1 $${{F}}_{{L}}=\frac{{{f}}_{{L}}{{\rho }}_{{f}}{{U}}_{{m}}^{2}{{a}}_{{p}}^{4}}{{{D}}_{{h}}^{2}}$$

2 $${R}{{e}}_{{C}}=\frac{{{\rho }}_{{f}}{{U}}_{{m}}{{D}}_{{h}}}{{\mu }}$$

where *ρ*_*f*_, *U*_*m*_ and *µ* are the fluid density, maximum velocity and dynamic viscosity, respectively. The lift coefficient *f*_*L*_ is dependent on the particle’s position in the channel, the channel Reynolds number, and the aspect ratio of the channel. In most practical applications of microfluidic chips (*Re* < 100), an average *f*_*L*_ value of 0.5 is employed to simplify the estimation^24^. Recently, it has been found that the lift coefficient is associated with the particle position in the channel, which allows Eq. (1) to be simplified based on the position of the particles. The net lift force near the channel center is simplified as

3 $${{F}}_{{L}}=\frac{{{f}}_{1}{\rho }{{U}}_{{m}}^{2}{{a}}_{{p}}^{3}}{{{D}}_{{h}}}$$

and becomes

4 $${{F}}_{{L}}=\frac{{{f}}_{2}{\rho }{{U}}_{{m}}^{2}{{a}}_{{p}}^{6}}{{{D}}_{{h}}^{4}}$$

near the channel walls^25^.

Particle migration can be further controlled by using curved channels^1^. The fluid passing through a curved passage is subjected to a radially outward centrifugal acceleration, and two counterrotating vortices are formed in the upper and lower halves of the passage, which are called Dean vortices. The magnitude of these secondary flows is quantified by a dimensionless Dean number (*De*) given by^26^.

5 $${De}=\frac{{\rho }{{U}}_{{f}}{{D}}_{{h}}}{{\mu }}\sqrt{\frac{{{D}}_{{h}}}{2{R}}}={Re}\sqrt{\frac{{{D}}_{{h}}}{2{R}}}$$

where *U*_*f*_ is the mean fluid velocity and *R* is the radius of curvature of the channel. In addition to the inertial lift force, particles in a curved channel are also affected by the centrifugal force and Dean drag force due to the channel curvature and the introduction of the Dean flow. However, the particle density used in most experiments is close to or slightly smaller than the density of the surrounding fluid, and the centrifugal force can be ignored^10^. Therefore, it can be approximated that the particles migrate to an equilibrium position under the combined action of the Dean drag force and inertial lift force. The maximum value of the Dean drag force (*F*_*D*_) can be estimated by the Stokes drag

6 $${{F}}_{{D}} \sim {\rho }{{U}}_{{m}}^{2}{{a}}_{{p}}{{D}}_{{h}}^{2}{/}(2{R}){\rm{.}}$$

In addition, the most distinctive characteristic of serpentine channel flow is that the direction of the Dean flow keeps alternating. Recent comprehensive reviews about particle behavior studies in inertial microfluidics can be found elsewhere^27^. The influence of the channel structure on the three-dimensional position of particles and the magnitude of the dominant force acting on the particles will be discussed in detail later.

## Results and Discussion

### Model verification

**Figure 1:**
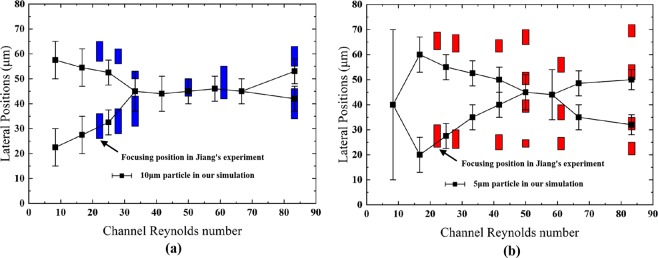
Comparison between our simulation and Jiang’s experiments (adapted from ref.^23^, permission of RSC Advances) for the particle focusing positions and focusing width in the serpentine channel (**a**) for the 10 μm particles and (**b**) the 5 μm particles. The plotted error bars indicate the width of the focusing positions.

A computational model was developed, and the results were compared with the experimental results of Jiang *et al*.^23^ to verify the reliability of the model. As specified in their experiments, a serpentine channel with a rectangular cross-section (80 µm × 40 µm) was studied. The flow field was simulated using COMSOL Multiphysics. Since the relationship between the driving force and *Re*_*C*_ is calculated from Poiseuille’s law, which is defined for a straight channel, *Re*_*C*_ in the simulation is higher than the actual value. In the particle distribution comparison for the simulation and experiments, *Re*_*C*_ of the simulation was divided by 1.5. A comparison between the simulation and experimental results for large particles (10 μm) is shown in Fig. 1a. The simulated focusing positions basically match the experimental results except that the focusing positions on one side slightly deviate at a small Reynolds number. The percentage of the estimated deviation was obtained by comparing the distance between the values from the simulation and experiments with the channel width. For large particles, the maximum deviation of the focusing position does not exceed 10%, and the average deviation of the focusing width is 4%, which is acceptable. The simulation results for small particles are also in line with the experimental trend (Fig. 1b), but small particles in the experiment are closer to the sidewalls than those in the simulation. Before small particles appear at the channel center, they focus well along the sidewalls. When the majority of small particles are focused at the channel center at higher *Re*_*C*_, a small portion of particles are focused closed to the sidewalls, which causes an increase in the deviation in the focusing position. These small particles may still be trapped by *F*_*L*_ when the flow velocity is relatively low (*Re*_*C*_ = 41.7), leading to a large deviation of approximately 18%. When *F*_*D*_ is the dominant force in the channel (*Re*_*C*_ = 61.1, *Re*_*C*_ = 83.3), these small particles may flow in the channel vertical center plane and be pushed by the Dean flow further to the sidewalls. Hence, relatively large deviations at higher *Re*_*C*_ can still be observed. Nevertheless, our model can predict the particle behavior and the focusing pattern in a fairly consistent manner, except for individual cases.

### Lateral focusing for various channel Reynolds numbers

**Figure 2:**
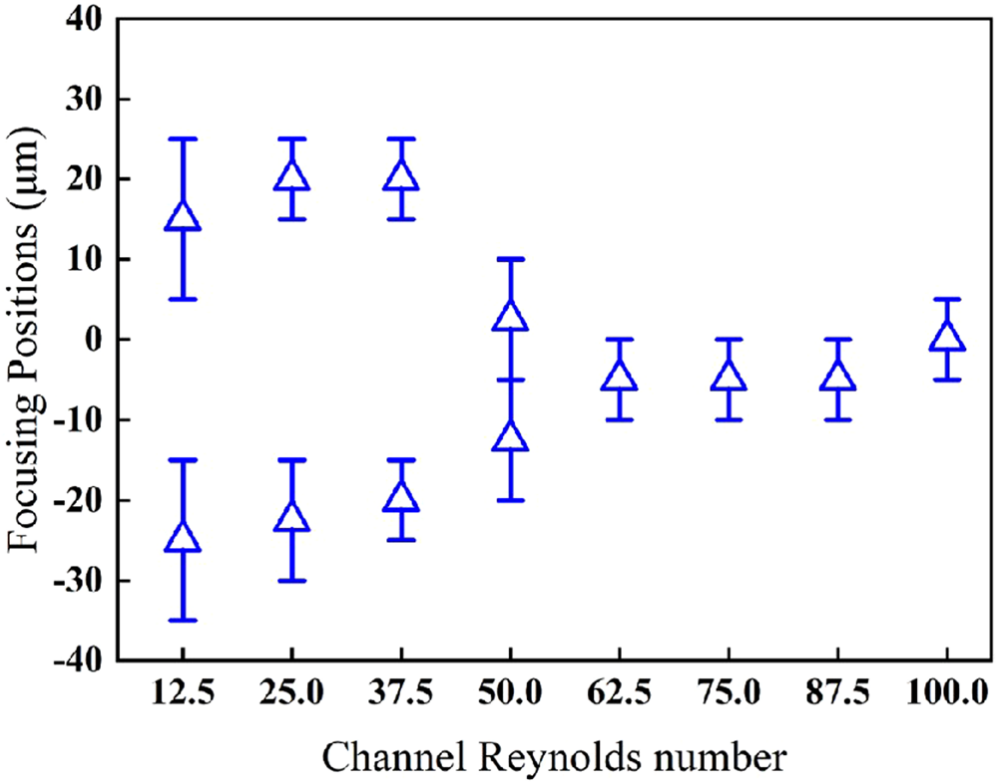
The lateral focusing positions of the 10 μm particles in the zigzag channel for different channel Reynolds numbers.

First, the trajectories of 10 μm particles (*λ* = 0.189) in the zigzag channel were simulated. The lateral focusing positions of the particles at the outlet are shown in Fig. 2. The data of the focusing positions and error bars for the particles correspond to mean and standard deviation values. As the channel Reynolds number (*Re*_*C*_) increases from 12.5 to 100 (corresponding to the flow velocity increasing from 15 cm/s to 120 cm/s), several particle focusing patterns can be observed in the channel. At a lower *Re*_*C*_ (*Re*_*C*_ = 12.5–37.5), the particles are focused along both sides of the channel. It can be considered that the dominant inertial lift force results in a two-sided focusing phenomenon, because at *Re*_*C*_ = 37.5, the outer edges of the particle focusing regions are approximately 15 μm (19% of the channel width) away from the sidewalls of the channel, which is similar to the reported straight channel inertial equilibrium positions^28^.

At a higher *Re*_*C*_ (*Re*_*C*_ > 62.5), the particle focusing positions tend to be stable with a width of approximately 12 μm, which is close to the particle diameter. Therefore, it can be considered that when *Re*_*C*_ is greater than 62.5, the particles exhibit a stable focusing state in which individual particles are sequentially arranged. This is because the Dean drag force is much larger than the inertial lift force and dominates the particle migration.

When *Re*_*C*_ ranges from 37.5 to 62.5, the particles are in a transition region. In this region, the inertial lift force and the Dean drag force are comparable and compete with each other. The particles occupy the area between the two focusing positions and tend to gradually move close together. The focusing position will approach the channel center symmetrically as *Re*_*C*_ increases and eventually form a stable single focusing position, as discussed above. All the simulations in this paper presented a similar tendency with varying *Re*_*C*_.

It is concluded that the simulated channel Reynolds number range can be divided into three regions: (I) a region dominated by the inertial lift force, (II) a transition region, and (III) a region dominated by the Dean drag force. However, the *Re*_*C*_ ranges of the three regions will differ for different curved channels and particle sizes.

### Particle migration characteristics

**Figure 3:**
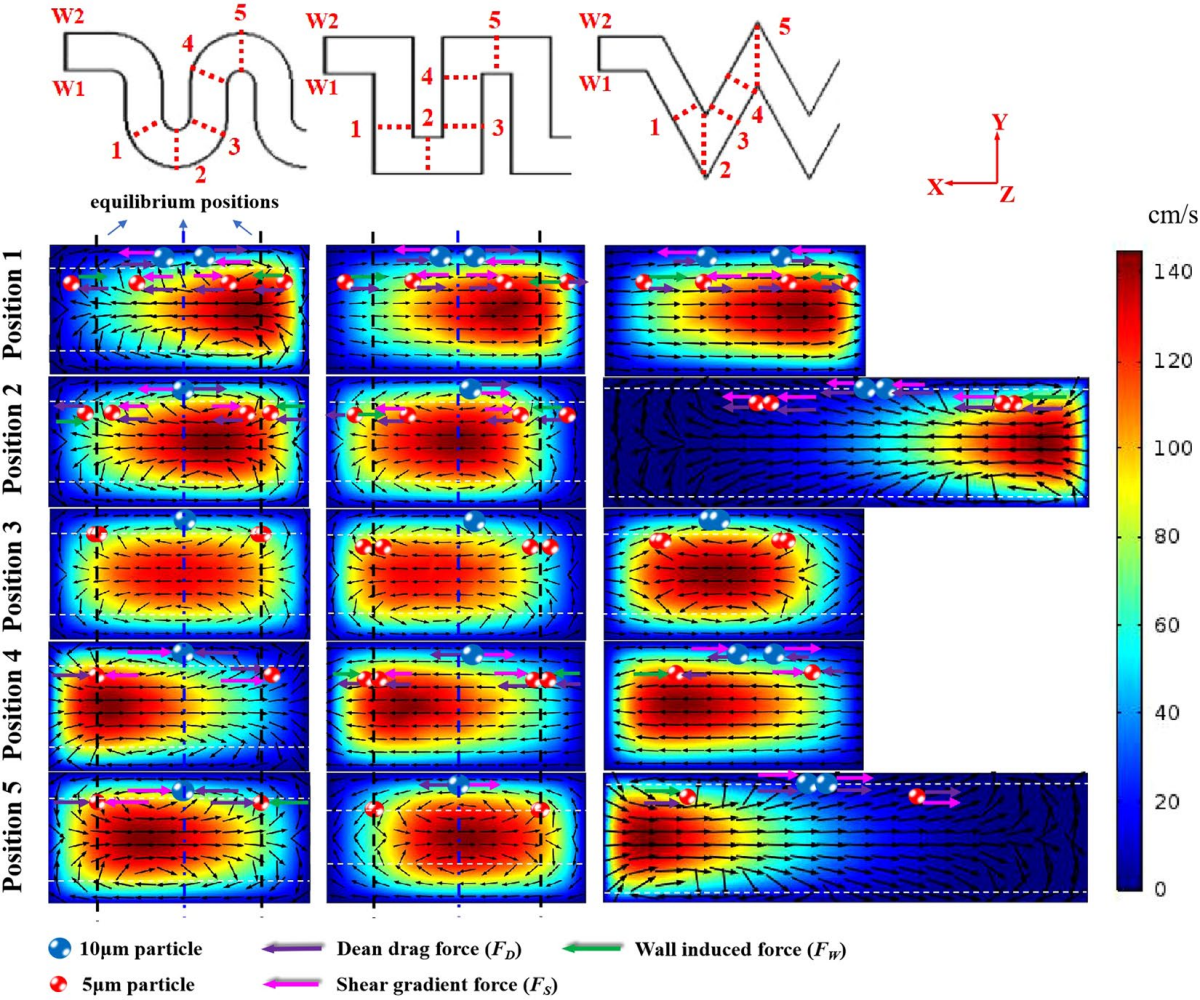
The cross-sectional view of the fluid field at five positions for Re_C_ = 50. The left side of the picture is W1, and the right side is W2. The white dotted line represents the zero Dean velocity line. The arrow size of the velocity vector in the figure is normalized.

The migration behavior of the particles is the result of the inertial lift force and Dean drag force in the flow field. To clarify the competition between these two forces, the flow patterns of several cross-sections in three curved channels were obtained, as shown in Fig. 3. It is clear that the flow patterns are symmetrical in the vertical direction due to the lack of a curvature variation in the channels in this direction. Thus, only particles in the upper half of the channel are marked in the figure. Observing position 1 of all three channels, the 10 μm particles (blue dots) are subjected to a large inertial lift and are found on both sides of the channel centerline. Because of the shear velocity gradient, the particles will first migrate in the vertical channel direction and then in the horizontal direction. In this case, the vertical positions of the 10 μm particles are supposed to be outside the zero Dean velocity line at the top and bottom of the channel. Moreover, the 5 μm particles (red dots) occupy four characteristic positions in the channel: two positions are closed to the wall regions, where *F*_*W*_ is directing the particles to the channel center, and the another two positions are close to the central region, where *F*_*S*_ is directing the particles to both sides of the channel. Since *F*_*S*_ is caused by the parabolic velocity profile in the channel center and is proportional to the particle size (see Eq. (3)), the vertical component of *F*_*S*_ acting on the 5 μm particles is weaker than that on the 10 μm particles. Therefore, the vertical positions of the 5 μm particles are between the two zero Dean velocity lines. Subsequently, the particles have different behaviors due to the different channel structures.

In the serpentine channel in Fig. 3, due to the constant change of the channel curvature, there are always two counterrotating vortices in the top and bottom halves of the channel. This makes the particle focusing process relatively stable. When the particles reach position 1, the velocity maximum is close to W2. The direction of the horizontal component of *F*_*S*_ is from the velocity maximum to the channel walls, and *F*_*D*_ is directed to W1 (−). The direction from W1 to W2 is the positive direction, indicated by “+”, and the opposite direction is the negative direction, indicated by “−”. The lateral position of the 10 μm particles moves towards the channel center, where *F*_*S*_ and *F*_*D*_ are balanced. With the joint effect of *F*_*W*_ and *F*_*D*_, the 5 μm particles near the channel walls rapidly migrate to the nearby equilibrium positions. For particles in the central region of the channel, the direction of *F*_*D*_ changes along the streamlines, in some positions helping *F*_*S*_ to accelerate the particles to the equilibrium and in other positions competing with *F*_*S*_ to disturb the particles from their equilibrium. When the particles reach position 2, the serpentine channel smoothly transitions as the particles pass the corner. The velocity maximum is close to the channel center, the direction of *F*_*S*_ is always from the velocity maximum to the two sidewalls of the channel, and *F*_*D*_ still maintains the previous direction (−). Due to the large Dean drag force in the alternating turns and the effect of the inertial lift force, the particles begin to migrate to the final equilibrium position. When the particles reach position 3 (the transition region), the velocity maximum has moved to the channel center, leading to no lift. Due to the abrupt change in the direction of the curvature, the curvature effect can be ignored. Thus, the Dean drag force becomes insignificant (see Eq. (6)). At this point, both the 5 μm and 10 μm particles have reached equilibrium positions and follow the streamlines until they reach the new velocity profile region. It should be noted that after position 3, the direction of the curvature changes, and whenever the curvature changes, the directions of the forces are opposite. At position 4, the change in the curvature causes the velocity maximum at the channel center to move to the opposite side. The direction of *F*_*D*_ also changes to W2 (+), and the particles swing near the equilibrium positions before *F*_*D*_ and *F*_*S*_ reach equilibrium again.

In the square wave channel, when the particles reach position 1, the particles exhibit similar motion to that in serpentine channels. The difference is that since the square wave channel does not have continuous curvature but is a combination of short straight channels, the velocity vector at position 1 only has a single direction towards W2. When the particles pass through a 90° corner and reach position 2, the direction of *F*_*D*_ is towards W1 (−). *F*_*D*_ increases due to the expansion of the cross-section, and *F*_*L*_ decreases. This change will break the balance between *F*_*D*_ and *F*_*L*_ and cause the particles to fall into the Dean vortex controlled by *F*_*D*_. After passing two corners, the particles reach position 3, and the direction of *F*_*D*_ remains towards W1 (−). This situation is the same as in the serpentine channel; both the 5 μm and 10 μm particles migrate close to the equilibrium positions. At position 4, the velocity vector again becomes a single direction towards W1.

In the zigzag channel, when the particles reach position 1, the motion of the particles is similar to that in the other two channels, and the velocity vector is in a single direction towards W2. Similar to the square wave channel, the zigzag channel has a straight structure after each corner, which can be regarded as a short straight channel. At the beginning of the short straight channel (position 3 in Fig. 3), the velocity vector will inherit the pattern from the previous corner (two counterrotating vortices). Then, the Dean vortex becomes smaller and eventually forms a single-direction vector (positions 1 and 4 in Fig. 3) similar to the inertial lift in the straight channel until the next corner. With the increase in *Re*_*C*_, the particles can pass through the straight region rapidly, and the Dean drag force will dominate. Therefore, the zigzag channel and the square wave channel can perform better under a high Reynolds number. When the particles reach position 2, the zigzag channel has a 60° corner, and the width of the cross-section is almost doubled. When the cross-section width increases, *D*_*h*_ will inevitably increase. According to Eqs (3) and (4), *D*_*h*_ is proportional to *F*_*D*_ but inversely proportional to *F*_*L*_. The velocity maximum is close to W2, and the direction of *F*_*D*_ is towards W1 (−). Both the 5 μm and 10 μm particles migrate towards W1 under the joint action of the large *F*_*D*_ and *F*_*L*_. It is worth noting that the spacing of the equilibrium positions on both sides of the 5 μm particles is enlarged. At position 3, both the 5 μm and 10 μm particles reach equilibrium positions and follow the streamlines until they reach the new velocity profile region. When the particles reach position 4, the velocity maximum is close to W1. Due to the reduction in the Dean drag force, the inertial lift force dominates, so both the 5 μm and 10 μm particles are offset towards W2. Position 5 is the mirror image of position 2, and the direction of *F*_*D*_ is directed towards W2 (+). The final effect is that the secondary flow sweeps small particles towards two-sided walls, facilitating the migration of particles towards the inertial equilibrium positions. Large particles are gradually focused near the centerline of the channel under the action of the large *F*_*D*_ and *F*_*L*_ at the corner. Then, a new cycle begins, and the particles will reach a stable equilibrium after a few cycles.

**Figure 4:**
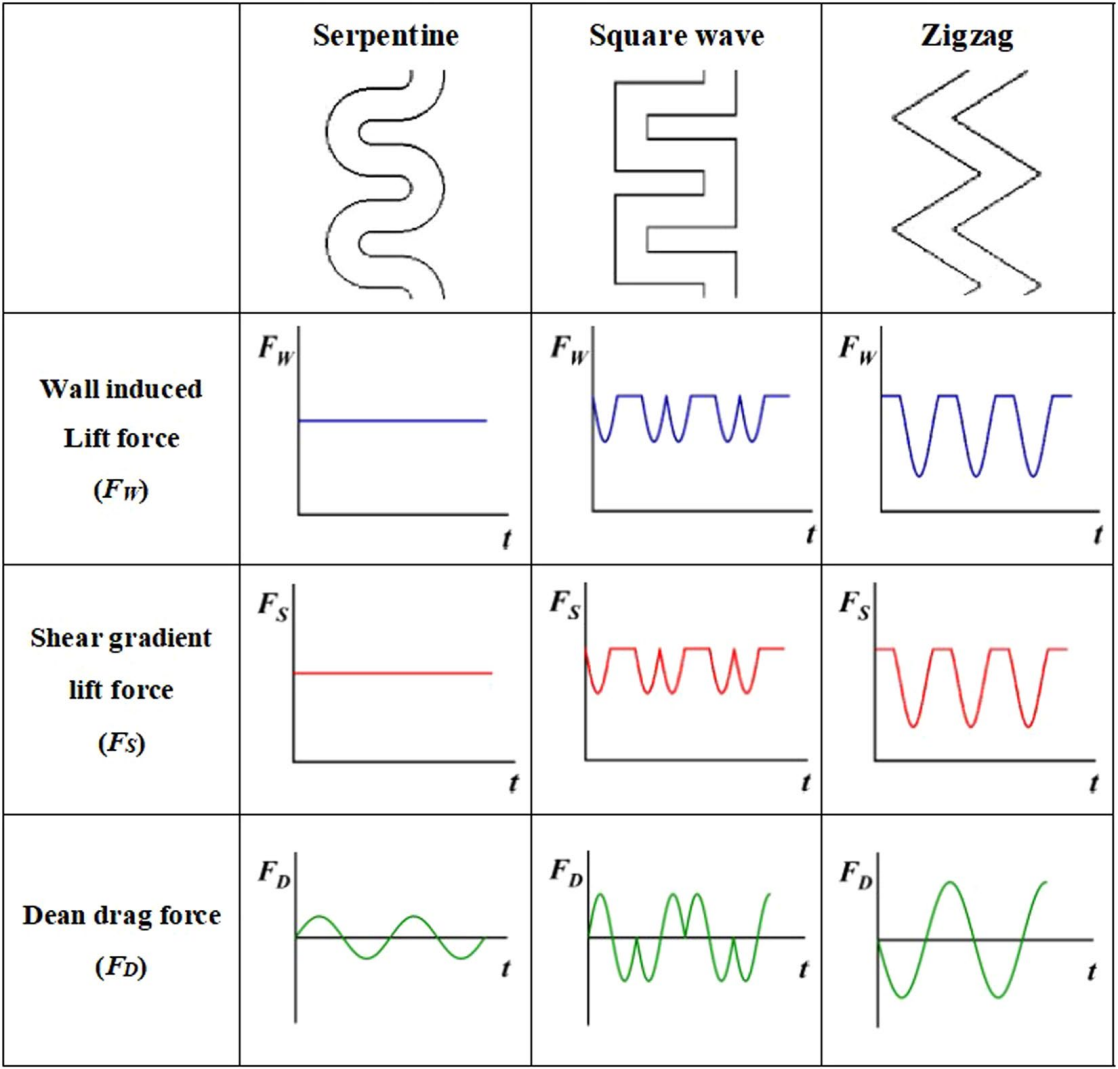
Channel geometries and force variation (*t* represents the position in the channel after a period of time).

To further investigate the difference in the forces acting on the particles, the force variations for the three channels are plotted in Fig. 4. The relationship between the inertial lift force and the hydraulic diameter at different positions in the channel are given by Eqs (3) and (4). Equation (3) is the inertial lift force at the channel center, which can be considered as a shear gradient lift force, and Eq. (4) is the inertial lift force at the channel wall, which can be considered as a wall-induced lift force. Therefore, Eqs (3) and (4) can be used to obtain the variation in *F*_*S*_ and *F*_*W*_ for different channel structures. The directions of *F*_*W*_ and *F*_*S*_ are fixed, so only the trends of their changes are shown in Fig. 4. As the channel cross-section width increases, *F*_*W*_ and *F*_*S*_ decrease. In addition, the relationship between the Dean drag force and hydraulic diameter obtained by Eq. (6) is also shown in Fig. 4. The difference is that the direction of the Dean drag force is indicated by positive (+) and negative (−) values of the Dean drag force, which is derived from the discussion of the velocity vector. When the channel cross-section increases in size, *F*_*D*_ also increases. However, the lift coefficient of these forces varies at every position of the cross-section; hence, it is difficult to obtain the value of the forces. Only the general trends of the forces along the channel are presented in Fig. 4. In the serpentine channel, there is no change in the cross-section, so *F*_*W*_ and *F*_*S*_ remain stable, and *F*_*D*_ changes direction due to the channel curvature changes. In the square wave channel, as the particles pass the corner, the cross-section increases in size, *F*_*D*_ increases, and *F*_*W*_ and *F*_*S*_ decrease. After entering the straight section, the cross-section returns to its original size. Therefore, *F*_*W*_ and *F*_*S*_ will gradually increase and then remain stable, while *F*_*D*_ will gradually decrease until the next corner. The development of the force competition in the zigzag channel is similar. However, compared to the square wave channel, the zigzag channel has a greater cross-section extension, and thus the forces exerted on the particles vary more dramatically.

### Particle focusing performance in different curved channels

It can be found from the previous discussion that the introduction of the Dean flow does not directly produce particle focusing but acts on the particles together with the inertial lift force to reduce the number of equilibrium positions. The superposition of these two forces is different in different curved channels. To explain the difference, the ratio of the magnitudes of *F*_*L*_ to *F*_*D*_^29^ was given as

7 $$\frac{{{F}}_{{L}}}{{{F}}_{{D}}} \sim \frac{1}{{\delta }}{(\frac{{{a}}_{{p}}}{{{D}}_{{h}}})}^{3}{R}{{e}}_{{C}}^{{n}},\,({n} < 0)$$

The curvature ratio is ${\delta }={{D}}_{{h}}{/}2{R}$. This relationship suggests that the ratio of *F*_*L*_ to *F*_*D*_ is strongly dependent on the ratio of *a*_*p*_ to *D*_*h*_. For the same *Re*_*C*_, small particles cannot be focused even when the channel is long enough due to the dominant role of *F*_*D*_, while large particles will be focused quickly.

**Figure 5:**
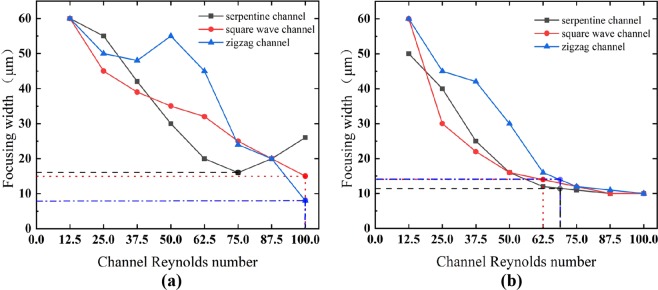
The relationship between the particle focusing width and the channel Reynolds number for the three curved channels for the (**a**) 5 μm particles and (**b**) 10 μm particles. The dotted line corresponds to the critical channel Reynolds number for particle focusing.

The competition between *F*_*L*_ and *F*_*D*_ determines the focusing pattern of particles of different sizes. The variations in the particle focusing width for different channels are shown in Fig. 5. It can be found that for the 5 μm particles (Fig. 5a), when *Re*_*C*_ ≤ 37.5, the particle focusing width in the square wave channel is 5–10 μm narrower than that of the other two channels for the same channel Reynolds number. When *Re*_*C*_ is increased to 50 and 75, the inertial lift force has a relatively slight influence on the particle migration in the vertical direction, the particles horizontally swing across the cross-section by the Dean drag force, and the focusing width gradually decreases. However, there are exceptions: the particle focusing width in the zigzag channel suddenly increases at *Re*_*C*_ = 50. This phenomenon results from the increases in *F*_*D*_ due to the extension of the channel cross-section at the corner, as mentioned above. Therefore, the spacing of the equilibrium positions on both sides of the 5 μm particles is enlarged due to the joint action of the large *F*_*D*_ and *F*_*L*_, which will suddenly broaden the focusing width. Although the square wave channel also has an extension of the channel cross-section, the scale of the extension is small. In Fig. 5, the focusing width is slightly larger only at *Re*_*C*_ = 62.5. When *Re*_*C*_ exceeds 75, the trend of the curve for the serpentine channel is different from that for the other two channels. In the serpentine channel, particles begin to spread, and the strong Dean flow drags particles to the sidewall again. The optimal focusing state is achieved at *Re*_*C*_ = 75. A similar phenomenon cannot be observed for the zigzag and square wave channels. The particles in these channels are still well focused, and the optimal focusing state is achieved at *Re*_*C*_ ≥ 87.5. In comparison, the particles in the zigzag channel are arranged more compactly. It is predicted that particle dispersion will occur at a value of *Re*_*C*_ that is large than our simulated *Re* range due to strong Dean flow. In summary, for the 5 μm particles, the zigzag channel is the best choice for a relatively high *Re*_*C*_ range, and the serpentine channel is also a good choice for moderate values of *Re*_*C*_.

The 10 μm particles exhibit similar behaviors in the different channels, as shown in Fig. 5(b). When *Re*_*C*_ is as large as 100, the 10 μm particles in all three channels are well focused. At a low *Re*_*C*_, the particles flow along the two sidewalls under the superposition of *F*_*L*_ and *F*_*D*_. If *Re*_*C*_ continues to increase, the particles will be focused at the channel center by the strong direction alternation of *F*_*D*_ (see Fig. 2). When *Re*_*C*_ exceeds 50, the focusing width curves of the three curved channels almost overlap. When the channel Reynolds number is high, the dominant effect of *F*_*D*_ on large particles is far greater than that of *F*_*L*_, and a single focusing position can be achieved along the channel center. With the increase in *Re*_*C*_, the migration of the 10 μm particles from both sides to the center is much faster than that of the 5 μm particles. Therefore, it can be seen that the size difference also plays an important role in the focusing pattern, and the large particles are more likely to be focused at the horizontal center of the channel. This is due to the contribution of both *F*_*L*_ and *F*_*D*_. On one hand, large particles are slightly closer to the channel center than small particles in the inertial straight channel because of the difference in *F*_*L*_; on the other hand, *F*_*D*_ has more influence than *F*_*L*_ in terms of a greater effect on large particles than small particles. It also can be seen from Fig. 5 that the focusing width decreases sharply as the channel Reynolds number increases, and focusing is achieved after a critical value (defined as the critical channel Reynolds number for particle focusing, *Re*_*CC*_). An exception is that the 5 μm particles in the serpentine channel are focused at *Re*_*C*_ = 75 and then diverge at a higher value of *Re*_*C*_. It should be noted that the *Re*_*CC*_ values of the 10 μm particles are all smaller than those of the 5 μm particles for the same channel. To comprehensively evaluate the focusing performance, the *Re*_*CC*_ data for all of the conditions studied here are listed in Table 1. The large 10 μm particles can be focused within a wide range of *Re*_*C*_ (>~60) in all three channels. The threshold of the serpentine channel is 62.5, a relatively lower value. Nevertheless, for the 5 μm particles, focusing becomes more difficult than for the large particles. In the serpentine channel, only one *Re*_*C*_ (=75) value leads to focusing; in the square wave and zigzag channels, the small particles are focused because the focusing width still decreases at *Re*_*C*_ = 100. However, the small particles do not reach the desired complete focusing state because of the limitation of the Reynolds number in this study; thus, the *Re*_*CC*_ values of the small particles in the square wave and zigzag channels are tentatively set to 100. If the Reynolds number is extended in a later study, a more accurate *Re*_*CC*_ value could be obtained.

**Table 1:** Critical channel Reynolds number required to achieve focusing.

	Critical channel Reynolds number *Re*_*CC*_		
	Serpentine	Square wave	Zigzag
5 μm particle		~100	~100
10 μm particle	62.5	68.5	68.5

**Figure 6:**
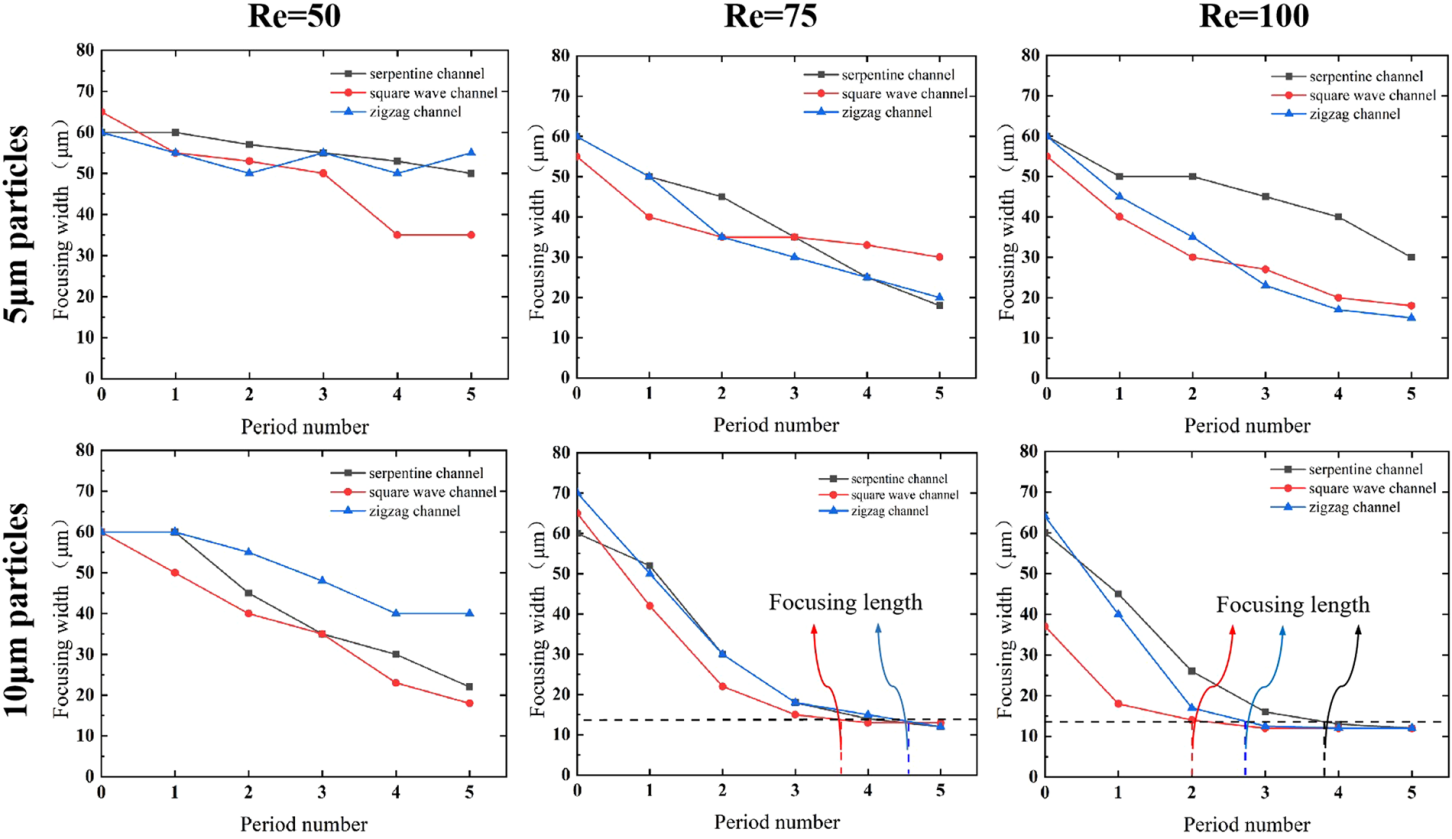
The focusing width of particles of different sizes after each structural period.

Another criterion for evaluating the focusing performance of a curved channel is the focusing length. To compare the focusing length under various conditions, the focusing widths of the 5 μm and 10 μm particles after each structural period are plotted in Fig. 6. A critical advantage of a small focusing length is the reduction in the fluidic resistance. The horizontal dotted line indicates that the focusing width tends to be stable, and the particles can be considered to be fully focused. As shown in Fig. 6, the 5 μm particles tend to focus only after the fifth period within the simulated period numbers, and a stable focusing width cannot be observed. However, the 10 μm particles achieve a stable focusing width for all three channels at *Re*_*C*_ = 75 and 100. Under the same conditions, the large particles are subjected to a greater inertia lift force and are less susceptible to interference by the Dean drag force, so they can achieve focusing more quickly than the small particles. This is consistent with the results of the critical channel Reynolds number. When *Re*_*C*_ = 75, the curves for the 10 μm particles in the serpentine channel and the zigzag channel almost overlap, and the particles in the channels are focused after the fourth period. The particles in the square wave channel are focused after only three periods, with a focusing length of approximately 1.5 mm. When *Re*_*C*_ = 100, the focusing length of the particles for the three channels is shortened to varying degrees. The square wave channel is still superior in terms of the focusing length. Hence, it can be concluded that the large particles can be focused within a shorter focusing length when the flow condition (*Re*_*C*_) is constant, and the square wave channel has advantages over the other two channels for the 10 μm particles due to the rapid focusing.

### Separation possibilities for different curved channels

**Figure 7:**
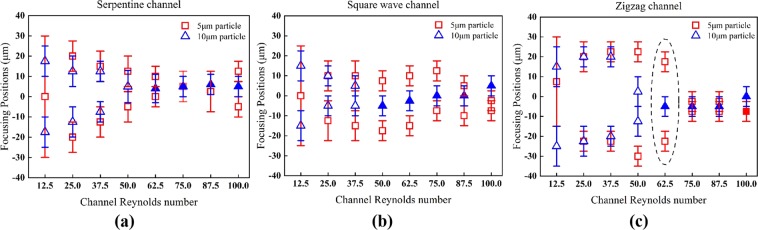
Focusing positions of the 5 μm (red□) and 10 μm (blueΔ) particles in the three curved channels for various channel Reynolds numbers. The plotted error bars indicate the width of the focusing positions, and the solid legend represents the single point focusing particles.

An interesting result of the above focusing characteristics is that the arrangement of particles of different sizes varies across the width of the channel. To show the distribution of particles intuitively, the overlaid *Re*_*C*_ maps in Fig. 7 graphically show the focusing positions and their deviations for the two sizes of particles for the three curved channels. The particle focusing positions are either distinct and able to be separated or overlapping and unable to be separated. Figure 7 shows that the smaller particles occupy both sides of the channel and that the particles have a wider region dominated by the inertial lift force. Correspondingly, the large particles are focused along the channel center, and the width gradually decreases to a single stable focusing line and thereby has a wider region dominated by the Dean drag force. The particles are focused along the channel center, and the width gradually decreases to a single stable focusing line. This contributes to the possibility of separating particles of two sizes.

In the serpentine channel (Fig. 7a), the regions of the 5 μm and 10 μm particles fully overlap. In the square wave channel (Fig. 7b) and the zigzag channel (Fig. 7c), the 5 μm particles migrate to the channel center more slowly than the 10 μm particles in the *Re*_*C*_ range of 50–75. The small particles line up on both sides of the large particles. There is a significant separation distance between the equilibrium regions of the particles of two sizes. In addition, in order to avoid difficulties in controlling the precision of the separation, it was found that the separation distance could not be too small. A distinct 10 μm distance can be observed at *Re*_*C*_ = 62.5 in the zigzag channel (dotted circle in Fig. 7c), which corresponds to the optimal separation condition in this study. When *Re*_*C*_ continues to increase to 100, the particles are both arranged in a line. The equilibrium regions of the two lines create a certain separation distance again, indicating a separation trend. It should be noted that the separation outlet system needs to be designed carefully for different separation behaviors, namely, three outlets for moderate *Re*_*C*_ values and two outlets for high *Re*_*C*_ values.

**Figure 8:**
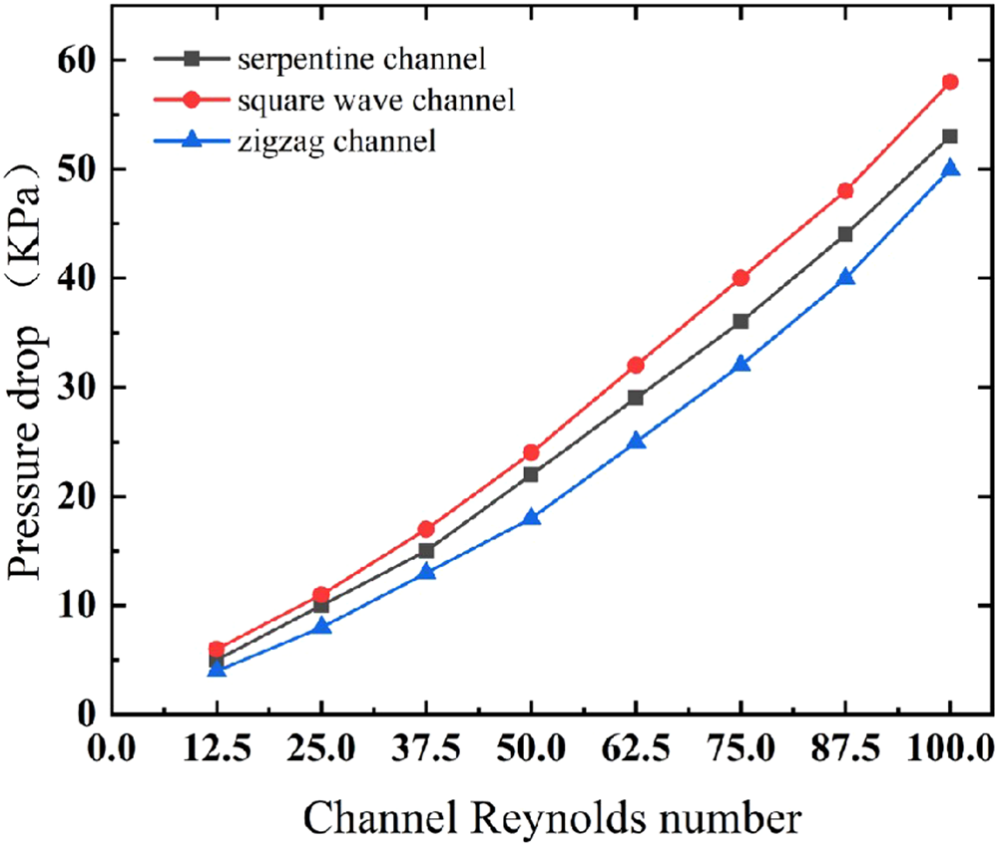
The fluid pressure drops corresponding to different Reynolds numbers in the three curved channels.

A high Reynolds number will lead to a large pressure drop, causing greater energy consumption and possible sample leakage. Thus, the pressure drop data were extracted to evaluate the channel performance, as shown in Fig. 8. The pressure drop of the zigzag channel generally exhibits a smaller pressure drop than the other two channels, which is on average 13.9% less than that of the serpentine channel and 22.7% less than that of the square wave channel. In other words, the overall performance of the zigzag channel is superior to that of the square wave and serpentine channels considering the focusing, separation and pressure drop.

## Conclusions

In this paper, a zigzag channel, which has not been used previously for inertial focusing studies, is compared to a serpentine channel and a square wave channel to explore their differences in terms of focusing effects and separation possibilities. From the particle trajectories and fluid fields in the curved channels, the competition between the inertial lift force and the Dean drag force exerted on the particles is illuminated clearly. The channel structures influence the flow velocity profile and the balance between *F*_*L*_ and *F*_*D*_, thus producing various focusing and separation behaviors. The results for the focusing width, focusing length and Reynolds number range show that, for small particle focusing, the zigzag channel is the best choice for a high *Re*_*C*_ value and the serpentine channel is the second choice for a moderate *Re*_*C*_ value. The three curved channels achieve a similar focusing performance for large particles and a high *Re*_*C*_, because the contribution of the Dean drag force is much greater than that of the inertial lift force. In addition, the particle distribution presents a distinct separation possibility. The optimal separation performance appears at *Re*_*C*_ = 62.5 in the zigzag channel with a separation distance of approximately 10 μm. Ultimately, the overall performance of the zigzag channel is superior to that of the square wave and serpentine channels considering the focusing, separation and pressure drop.

The zigzag channel has prospects in focusing and separation applications. Future research should consider finite-size effects in the modeling of the particles. In this research, the interaction between the particles was ignored, as the particles were individually released at each time step and experienced a weak interaction. Particle trains, which are seriously affected by particle interactions, may produce new phenomena in the flow pattern and particle migration. This is our ongoing work. Because of limitations in terms of code complexity and computing resources, it is challenging to obtain quantitative data from numerical simulations. Therefore, our long-term vision includes the development of viable simulation methods.

## Methods

**Figure 9:**
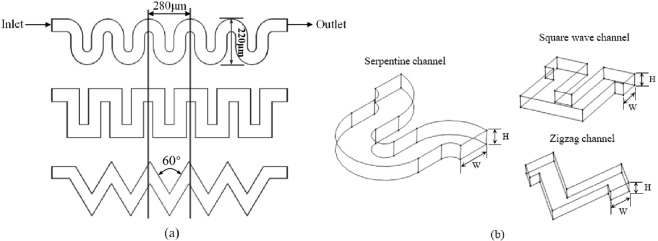
Schematic diagrams of the three curved channels: (**a**) top view of the three curved channels and (**b**) unit structure of the three curved channels.

To analyze and compare the focusing behavior of the particles, the particle trajectories and fluid fields in three curved channels were simulated using the commercial computational fluid dynamics (CFD) software COMSOL Multiphysics 5.3 (Burlington, MA). Structural diagrams of the three curved channels studied in this paper are shown in Fig. 9(a,b) describes the structural unit of the channels. As shown in the figure, the length of the channel structural unit is 280 μm, and the channel height H and the width W are 40 μm and 80 μm, respectively. Thus, the channel depth-to-width ratio AR is 0.5. These channels were modeled and simulated by using tetrahedral meshes, and a mesh independence test was conducted. Taking the serpentine channel as an example, the grid number for different meshing methods ranged from 8.98 × 10^4^ to 3.77 × 10^5^, and 2.92 × 10^5^ was selected as the best grid number. The same test was conducted for the other two channels to obtain the best grid number. Each model was solved using the GMRES iterative solver. The element size and quality were adapted based on computer memory restrictions and the actual size of the model for each structure. The densities of the particles and water were both 1000 kg·m^−3^. The viscosity of water was 1 × 10^−3^ kg·m^−1^·s^−1^.

The full Navier-Stokes equations were solved by a laminar flow model using single-phase and incompressible flow assumptions. For an incompressible and steady laminar flow, the governing Navier-Stokes equations can be expressed as ${\rho }({\boldsymbol{u}}\cdot \nabla ){\boldsymbol{u}}=\nabla \cdot [\,-\,{p}{\boldsymbol{I}}+{\mu }(\nabla {\boldsymbol{u}}+{(\nabla {\boldsymbol{u}})}^{{T}})]+{\boldsymbol{F}}$ and ${\rho }\nabla \cdot {\boldsymbol{u}}=0$, where the symbols follow the default definition in COMSOL. Then, the particle tracking module was used to predict the particle trajectories in the curved channels, and the particle momentum was given by Newton’s second law. The particles experienced a drag force given by Stokes’ law, which includes a correction factor for the drag force for particles near walls. The drag force was expressed as ${{\boldsymbol{F}}}_{{D}}=6{\pi }{\mu }{{a}}_{{p}}({\boldsymbol{u}}-{\boldsymbol{v}})$, where *μ* is the fluid viscosity, ***u*** is the fluid velocity, and ***v*** is the particle velocity. The lift force was expressed as ${{\boldsymbol{F}}}_{{L}}={\rho }\frac{{{r}}_{{p}}^{4}}{{{D}}^{2}}{\beta }({\beta }{{G}}_{1}({s})+{\gamma }{{G}}_{2}({s})){\boldsymbol{n}}$, where *r*_*p*_ is the particle radius, ***n*** is the wall normal at the nearest point on the sidewall, *s* is the nondimensionalized distance from the particle to the sidewall, divided by *D* so that 0 < *s* < 1 for particles in the channel, and *G*_1_ and *G*_2_ are built-in functions of the nondimensionalized wall distance *s*. In addition, the fluid-particle interaction interface combines particle tracing for the fluid flow and laminar flow interfaces to model the motion of particles in a fluid. In this interaction interface, the acceleration or deceleration of particles creates a significant volume force that affects the motion of the fluid. The fluid-particle interaction node computes a volume force that is equal in magnitude and opposite in direction to the total drag force that the fluid exerts on the particles. Given an array of idealized point masses such that the position vector of the *i*th particle is denoted by ***q***_***i***_, the volume force at position ***r*** is ${{F}}_{{V}}({\boldsymbol{r}})={-}\mathop{\sum }\limits_{{i}=1}^{{N}}{{\boldsymbol{F}}}_{{D},{i}}{\delta }({\boldsymbol{r}}-{{\boldsymbol{q}}}_{{i}})$, where *δ* is the Dirac delta function, ***F***_*D*,*i*_ is the drag force exerted on the *i*th particle, and *N* is the total number of particles. Particles were added according to particle diameter and density and were considered as points. Different particle sizes only affected the magnitude of the force acting on the particles. One 5 μm spherical particle and one 10 μm spherical particle were randomly released at each time step. The particle release time ranged from 0 to 2 × 10^−4^ s, and the time interval was 9 × 10^−6^ s. The total number of released particles was 46, with equal numbers of 5 μm and 10 μm particles. For different flow rates, an appropriate time step was selected accordingly. For low flow rates, the required time step was smaller, because the particles moved slower than for a high flow rate. The time steps for different Reynolds numbers are shown in Table 2. The laminar inflow boundary condition was used to automatically compute a fully developed fluid velocity profile at the inlet boundary. Zero static pressure and nonslip boundary conditions were set at the channel outlet and channel wall boundaries, respectively.

**Table 2:** Time steps under different channel Reynolds numbers.

*Re*_*C*_	12.5	25	37.5	50	67.5	75	87.5	100
Time step	5 × 10^−6^	7 × 10^−6^	9 × 10^-6^	1.1 × 10^−5^	1.3 × 10^−5^	1.5 × 10^−5^	1.7 × 10^−5^	1.9 × 10^−5^
Total time	1.5 × 10^−2^	1.2 × 10^−2^	1 × 10^−2^	8 × 10^−3^	7 × 10^−3^	6 × 10^−3^	5 × 10^−3^	4 × 10^−3^
